# Blau syndrome with atrophoderma vermiculata-like appearance: a case report

**DOI:** 10.3389/fimmu.2026.1759464

**Published:** 2026-03-04

**Authors:** Jingwei Zheng, Jianfang Sun

**Affiliations:** 1 Hospital for Skin Diseases, Institute of Dermatology, Chinese Academy of Medical Sciences and Peking Union Medical College, Nanjing, China; 2 Department of Pathology, Hospital for Skin Diseases, Institute of Dermatology, Chinese Academy of Medical Sciences and Peking Union Medical College, Nanjing, China

**Keywords:** atrophoderma vermiculates (AV), Blau syndrome (BS), granulomatous inflammation, nucleotide oligomerization domain 2 (NOD2), whole-genome sequencing

## Abstract

We report a rare case of Blau syndrome in a 1-year-old boy. The patient presented with characteristic facial manifestations, notably skin lesions exhibiting atrophoderma vermiculates-like appearance; ocular and articular symptoms were notably absent at presentation. Histopathological examination confirmed non-caseating granulomatous inflammatory changes. Whole-genome sequencing (WGS) identified a heterozygous pathogenic mutation (p. Arg307Trp) in the nucleotide oligomerization domain 2 (NOD2) gene. Treatment with oral prednisone combined with topical vitamin E application resulted in a significant improvement of his skin lesions.

## Introduction

Blau syndrome (BS) is a rare autosomal dominant autoimmune disorder usually caused by mutations in the nucleotide-binding oligomerization domain 2 (NOD2)/CARD15 gene (1, 2). Herein we report the first diagnosis of BS in a patient presenting with distinctive atrophoderma vermiculata-like appearance with an uncommon NOD2 mutation (c.919C>T).

## Case presentation

**Figure 1:**
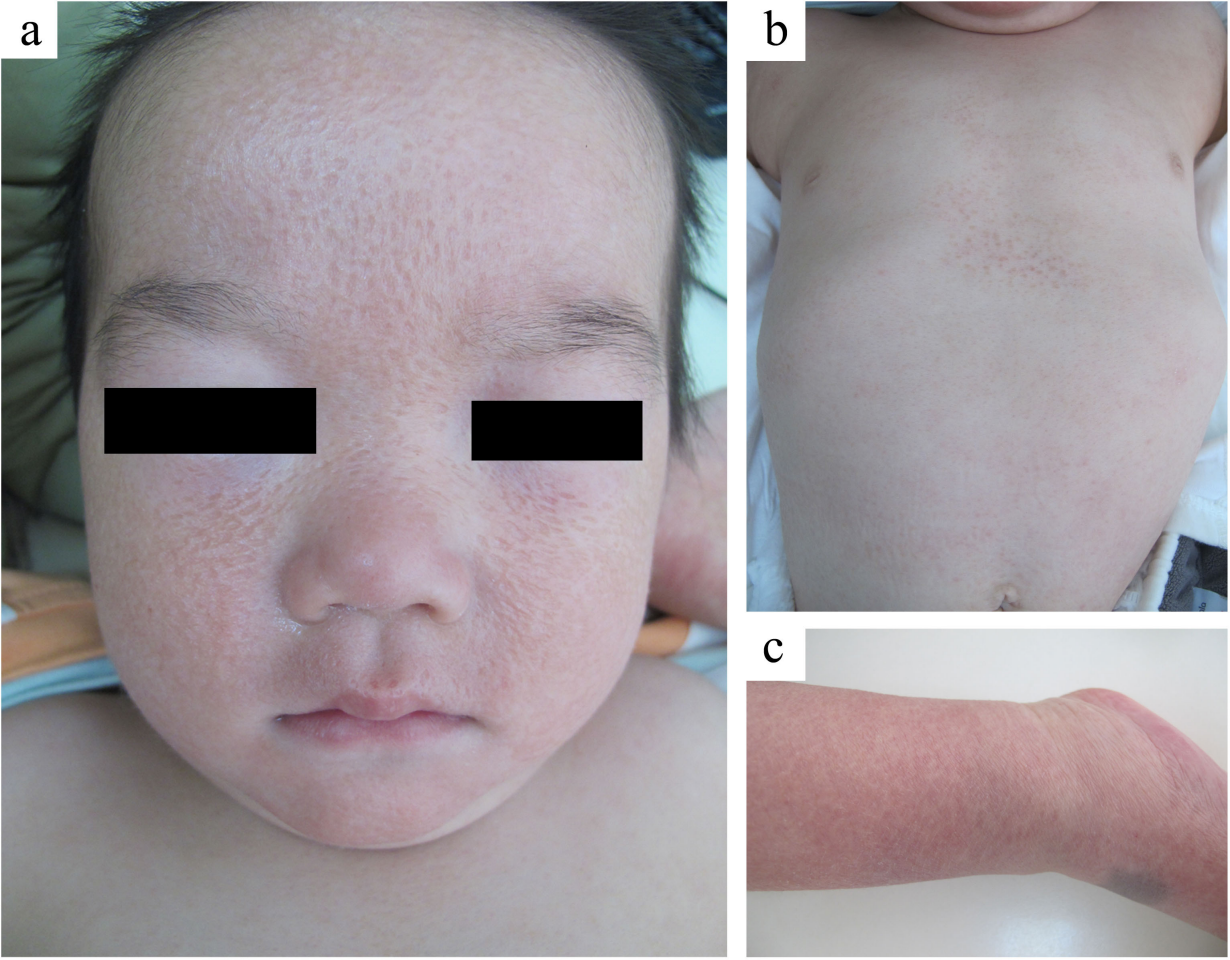
Clinical presentation images in the case. **(a)** Atrophoderma vermiculata-like appearance. **(b, c)** Milia-sized papules distributed on the trunk and limbs.

**Figure 2:**
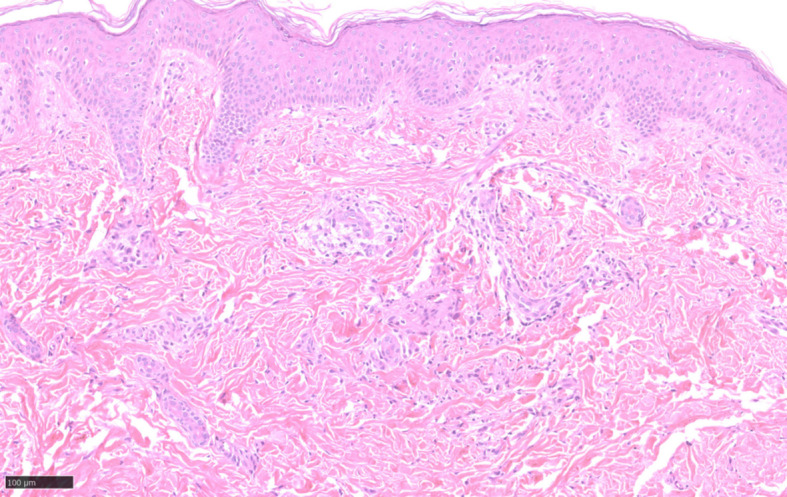
Hematoxylin-eosin (HE, ×20) staining showed a generally normal epidermis. Small vessels proliferated in the superficial dermis, with a small amount of lymphocyte infiltration around. Granulomatous inflammation was seen in the middle and upper dermis.

A 1-year-old boy was admitted to our hospital complaining of progressive facial vermiform atrophy with papules in the trunk and extremities. The condition dates back to him at 3 months of age (October, 2023) when the red papules are widespread all over the body. Initially, the local hospital considered the case to be Langerhans cell histiocytosis (LCH). The pathological findings from his trunk revealed focal histiocytic aggregates with multinucleated giant cells. However, immunohistochemistry (IHC) showed positivity for CD68 and Ki67 (<5%+), while S100, CD1a, CD117, and Langerin were negative, thereby ruling out LCH. However, the definitive diagnosis remained unclear. Topical low-potency steroids yielded a poor response with the facial lesion progressing to atrophy. In November 2024, the patient was transferred to our hospital for further diagnosis and therapy. Physical examination showed facial atrophoderma vermiculatas and milia-sized papules distributed on the trunk and limbs (**Figure 1**). Microscopic examination demonstrated a non-caseating granulomatous inflammation in the mid-to-upper dermis (**Figure 2**). These findings prompted a consideration of BS. Further evaluation of the child revealed no ocular or joint abnormalities or others, but his whole genome sequencing (WGS) analysis identified a pathogenic heterozygous NOD2 variant (p. Arg307Trp) which was inherited from his father with a history of ankylosing spondylitis. By integrating the evidences above, the diagnosis as BS was made. Low-dose oral prednisone (5 mg per day) and topical vitamin E cream were applied. As for the facial vermiform atrophy, surgical grinding or ablative fractional laser treatment may be suggested several years later. Follow-up revealed that, after 6 months of treatment, the skin lesions on the patient’s trunk and limbs completely subsided with no aggravation of the facial lesions. In January 2026, the patient returned for a follow-up visit due to recurrent red papules on the trunk. No abnormalities were found in the eye and joint examinations. The prednisone dosage was adjusted to 7.5 mg per day, and topical application of vitamin E and mucopolysaccharide polysulfate cream was prescribed.

## Discussion

BS is classically defined by the triad of granulomatous arthritis, uveitis, and cutaneous involvement (1, 2). However, in its early stage, the disease may present solely with cutaneous manifestations which can be subtle and considerably diverse, thereby being misdiagnosed LCH (3). In our case, however, based on the clinical manifestations, HE staining, and IHC, LCH was ruled out. Combined with NOD2 mutation, BS with atrophoderma vermiculata-like appearance was diagnosed.

The distinguishing feature of this case lies in the patient’s facial lesions which progressively developed atrophy, which once delayed our diagnosis. In other BS patients with ineffective treatment, lesions of lichenoid papules usually enlarge or coalesce into plaques rather than undergo atrophy. Similar atrophy can be a sequela of infantile acne following follicular papules, pustules, or nodules. Approximately 17% of infantile acne will develop atrophic scars (4). By contrast, atrophoderma vermiculatum (AV) is currently regarded as an agnogenic atrophic keratosis pilaris subtype resulting from an unclear inflammatory process, which typically manifests in childhood with erythema and follicular papules that evolve into densely distributed, small (1–3 mm) follicular depressions, which collectively present a “worm-eaten” appearance (5). Another rare idiopathic atrophic skin disease is atrophoderma of Pasini and Pierini (APP). However, the depressed skin lesions of this disease can reach several centimeters in diameter. It often begins on the trunk, especially at the back, and is more common in young women (6).

Based on the patient’s skin lesion and pathological findings, infectious granulomas and related inflammatory diseases were ruled out. However, when a patient has onset in childhood and has a NOD2 gene mutation, it is suggested to differentiate BS from other NOD2-related diseases, especially Crohn’s disease (CD) and Yao syndrome. CD is an immune-mediated inflammatory bowel disease, of which mutations in NOD2 are mainly located in the LRR domain. Patients of CD present with systemic symptoms such as fever, intestinal symptoms, and extraintestinal manifestations involving the eyes, joints, and skin. Its skin involvement usually presents with tender nodules, ulcers, or pustules, such as erythema nodosum and pyoderma gangrenosum (7). Yao syndrome is a recently proposed systemic autoinflammatory disease with NOD2 mutations detected in all its domains. Its main symptoms include periodic fever, gastrointestinal symptoms, arthritis, and skin lesions. The cutaneous manifestations commonly present as scattered individual or coalescing erythematous plaques throughout the body. Our patient did not exhibit systemic symptoms, denied history of gastrointestinal discomfort, and the skin lesions were inconsistent, therefore, the above two diagnosis was not considered (8).

BS-associated NOD2 mutations predominantly localize to NACHT domain, which included the NBD domain (AA215-445), HD1 domain (AA446-505), WHD domain (AA506-622), and HD2 domain (e.g., E667K, N670K, A755V);with fewer mutations identified in the another domain, LRR(e.g., Q809K) (9). An assessment for the structural and functional impacts of NOD2 missense mutations proposed six highly deleterious and destabilizing mutations located at conserved positions (10). A comprehensive comparative analysis in ophthalmic practice categorizes mutations into pathogenic and non-pathogenic groups (11). Patients harboring these pathogenic mutations present with more severe manifestations and reduced treatment responsiveness compared to individuals with non-pathogenic variants. This mutation-specific disease severity pattern may extend to dermatological manifestations (12), suggesting incomplete penetrance and variable expressivity in NOD2-related pathophysiology, although NOD2 mutations are considered gain-of-function variants with a disease of high penetrance (13). In support of this, Chang et al. and Saulsbury et al. independently reported BS cases harboring the C483W and E383K NOD2 mutations, respectively, each demonstrating incomplete penetrance (14). The NOD2 variant (p. Arg307Trp) in our case, located in a conserved site, is a pathogenic variant. This genetic and phenotypic variability may explain the unique progression of facial lesions to an atrophoderma vermiculatum-like appearance observed in the present case, distinguishing it from previously reported BS presentations. The varied skin lesions in different BS patients might be attributed to different mutation sites.

Therapeutic approaches for BS are currently tailored based on disease severity, with available medications ranging from glucocorticoids to biologics, such as anti-tumor necrosis factor-alpha drugs (15).

## Conclusions

We report the first diagnosis of BS in an infant patient presenting with atrophoderma vermiculata-like appearance. Clinical heterogeneity of BS can lead to challenges in early diagnosis or misdiagnosis, particularly in cases where the clinical presentation is atypical or incomplete. Early recognition and effective treatment of BS are crucial to prevent irreversible organ damage. The identification of skin lesions as non-caseating granulomatous in nature, coupled with genetic testing for NOD2 mutations, is essential to confirm the diagnosis in a timely manner. In terms of management, anti-inflammatory agents and biological therapies have shown promise in controlling the inflammatory process.

## Data Availability

The raw data supporting the conclusions of this article will be made available by the authors, without undue reservation.
